# Ultrasound assessment of the pelvic sidewall: methodological consensus opinion

**DOI:** 10.1002/uog.29122

**Published:** 2024-11-05

**Authors:** D. Fischerova, C. Culcasi, E. Gatti, Z. Ng, A. Burgetova, G. Szabó

**Affiliations:** ^1^ Gynecologic Oncology Centre, Department of Gynecology, Obstetrics and Neonatology, First Faculty of Medicine Charles University and General University Hospital in Prague Prague Czech Republic; ^2^ Dipartimento Scienze della Vita e Sanità Pubblica Università Cattolica del Sacro Cuore Rome Italy; ^3^ Department of Biomedical Science for Health University of Milan Milan Italy; ^4^ Department of Gynaecological Oncology KK Women's and Children's Hospital Singapore; ^5^ Department of Radiology, First Faculty of Medicine Charles University and General University Hospital in Prague Prague Czech Republic; ^6^ Department of Obstetrics and Gynaecology, Faculty of Medicine Semmelweis University Budapest Hungary

**Keywords:** anatomy, clinical cases, endometriosis, gynecological tumor, pelvic sidewall, transvaginal ultrasound

## Abstract

A standardized methodology for the ultrasound evaluation of the pelvic sidewall has not been proposed to date. Herein, a collaborative group of gynecologists and gynecological oncologists with extensive ultrasound experience presents a systematic methodology for the ultrasonographic evaluation of structures within the pelvic sidewall. Five categories of anatomical structures are described (muscles, vessels, lymph nodes, nerves and ureters). A step‐by‐step transvaginal ultrasound (or, when this is not feasible, transrectal ultrasound) approach is outlined for the evaluation of each anatomical landmark within these categories. Accurate assessment of the pelvic sidewall using a standardized approach improves the detection and diagnosis of non‐gynecological pathologies that may mimic gynecological tumors, reducing the risk of unnecessary and even harmful intervention. Furthermore, it plays an important role in completing the staging of malignant gynecological conditions. Transvaginal or transrectal ultrasound therefore represents a viable alternative to magnetic resonance imaging in the preoperative evaluation of lesions affecting the pelvic sidewall, if performed by an expert sonographer. A series of videoclips showing normal and abnormal findings within each respective category illustrates how establishing a universally applicable approach for evaluating this crucial region will be helpful for assessing both benign and malignant conditions affecting the pelvic sidewall. © 2024 The Author(s). *Ultrasound in Obstetrics & Gynecology* published by John Wiley & Sons Ltd on behalf of International Society of Ultrasound in Obstetrics and Gynecology.

## INTRODUCTION

The aim of this consensus opinion is to introduce a systematic methodology for evaluating the pelvic sidewall using ultrasound. Sonographic assessment of the anatomical structures within the pelvic sidewall plays a crucial role in the detection of metastatic spread of gynecological malignancies and provides treating specialists with necessary information prior to decision‐making. Evaluation of the pelvic sidewall with a precise methodological approach also plays a role in benign gynecological disease. This is especially true in the case of deep infiltrating endometriosis, in order to evaluate all possible locations of involvement, such as within the sacral plexus or the sciatic nerve^1^.

Surgically, the pelvic sidewall has three layers. The superficial layer contains the peritoneum and the ureter, the middle layer contains the branches of the internal iliac vessels and the deep layer includes muscles and branches of the sacral plexus, being in close contact with the pelvic bones. This consensus opinion aims to address five anatomical categories of the pelvic sidewall (muscles, vessels, lymph nodes, nerves and ureters) and to emphasize significant sonographic landmarks pertaining to this clinically significant region. In order to enhance comprehension of the subject matter, this consensus opinion is augmented by inclusion of supplementary videoclips (Videoclips [Link], [Link], [Link], [Link], [Link], [Link], [Link], [Link], [Link], [Link], [Link]), accessible online, which present the transvaginal ultrasound methodological approach to document each of these five categories (Videoclips [Link], [Link], [Link], [Link]), along with typical pathological findings (Videoclips [Link], [Link], [Link], [Link], [Link], [Link]). This study was performed in accordance with current Guidelines of Good Clinical Practice ICH‐GCP (ichgcp.net), the seventh revision of the Declaration of Helsinki, and applicable regulatory and country‐specific requirements.

## PELVIC SIDEWALL ANATOMY

**Figure 1:**
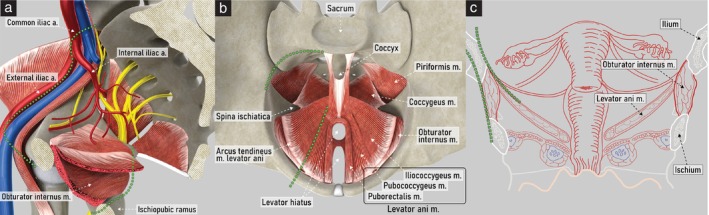
Pelvic sidewall boundaries. Schematic diagrams showing boundaries on right hemipelvis: (a) medial view, with superior and inferior boundaries indicated (green dotted lines); (b) superior view, demonstrating anterior and posterior boundaries (green dotted lines); and (c) coronal view, demonstrating medial and lateral boundaries (green dotted lines). a., artery; m., muscle.

The boundaries of the pelvic sidewall are as follows (Figure 1).

- Superiorly, it is bounded by the interiliac bifurcation, corresponding to the innominate line and the pelvic brim. The pelvic fasciae consist of two components: the endopelvic (parietal pelvic) fascia, which covers the pelvic brim and overlies the external iliac vessels and pelvic muscles, and the visceral fascia, which covers the pelvic organs and the supplying vessels and nerves. The visceral pelvic fascia also covers the connective tissue of the parametrium^2^.
- Inferiorly, it is bounded by the ischiopubic rami and the lower border of the obturator internus muscle.
- Medially, it is bounded by the pelvic fascia covering the levator ani muscle (composed of three striated muscles on each side: the iliococcygeus, pubococcygeus and puborectalis muscles).
- Laterally, it is bounded by the obturator membrane and the pelvic bones (pelvic aspects of the ilium and ischium).
- Anteriorly, it is bounded by the anterior border of the iliococcygeus muscle.
- Posteriorly, it is bounded by the sacroiliac joint.

## EXAMINATION METHODOLOGY

The pelvic sidewall should be assessed using a high‐frequency endocavitary probe. The patient lies in a dorsal lithotomy position and the bladder is ideally emptied prior to assessment. No routine preparation or patient fasting is required. The endocavitary probe can be inserted into either the vagina or the rectum. While, generally, a transvaginal approach is used, a transrectal approach is preferred in patients who are *virgo intacta*, have a stenotic vagina or have undergone brachytherapy. This approach is also recommended in patients with advanced vulvar, vaginal or cervical cancer, in whom the probe cannot be inserted easily into the vagina, mitigating the risk of bleeding from contact with friable tumor surfaces. Assessment of the pelvic sidewall can then commence in a stepwise, methodological fashion.

## ASSESSMENT OF PELVIC SIDEWALL

The ultrasound methodology of assessing the pelvic sidewall will be presented under the following subheadings: mobility, muscles, vessels, lymph nodes, nerves and ureters.

### Dynamic tests of mobility

A critical component of ultrasound assessment of the pelvic sidewall is to distinguish the structures belonging to the peritoneal cavity from those of the pelvic sidewall (Videoclip S1). Using dynamic evaluation by applying gentle pressure with the endocavitary probe, the mobility of individual structures is observed. Additionally, the operator can gently push simultaneously with both the free hand on the lower abdomen and the endocavitary probe. The displacement of intraperitoneal organs due to the applied pressure can be seen on ultrasound as a sliding movement^3^. Intraperitoneal organs are generally mobile when pushed with the endocavitary probe, whereas retroperitoneal organs are more fixed and show a lack of movement in most cases^4^.

### Muscles

#### Anatomy

**Figure 2:**
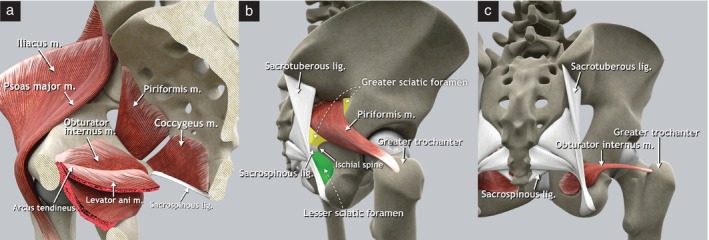
Pelvic sidewall muscles. (a) Schematic diagram of right hemipelvis, medial view. The arcus tendineus is a thickening of the fascia of the obturator internus muscle that serves as the origin of the iliococcygeus muscle. (b) Schematic diagram demonstrating insertion of piriformis muscle on apex of the greater trochanter of the femur and location of greater (yellow) and lesser (green) sciatic foramina (lateral view of right os coxae). The piriformis muscle divides the greater sciatic foramen into supra‐ and infrapiriform foramina. (c) Schematic diagram demonstrating insertion of obturator muscle onto the greater trochanter of the femur in dorsal view. lig., ligament; m., muscle.

The muscles of the pelvic sidewall are the obturator internus, levator ani, piriformis and coccygeus (Figure 2). The obturator internus muscle originates from the inferior margin of the superior pubic ramus and from the pelvic surface of the obturator membrane. Its tendon exits the pelvis through the lesser sciatic foramen to insert onto the greater trochanter of the femur. The obturator fascia covers the inner surface of the obturator internus muscle. The iliococcygeus muscle, which forms part of the levator ani, originates from the tendinous arch of the obturator fascia and the ischial spine, and inserts onto the anterolateral aspect of the coccyx, the iliococcygeal raphe and the anococcygeal ligament.

The (ischio)coccygeus muscle lies in continuity with the posterior part of the iliococcygeus muscle and, together, they form the pelvic diaphragm. It originates from the ischial spine and is inserted at the level of the sacrum (fifth sacral vertebra) and the coccyx^5^. The sacrospinous ligament attaches to the posterior surface of the coccygeus muscle^6^. The piriformis muscle originates from the anterior surface of the sacrum (between the second and the fourth sacral vertebrae), the upper margin of the greater sciatic notch and the capsule of the sacroiliac joint. Its muscle fibers traverse the greater sciatic foramen, exiting the pelvis, and insert onto the apex of the greater trochanter of the femur.

#### Ultrasound methodology (Videoclip S2)

#### First step: identify the obturator internus muscle

**Figure 3:**
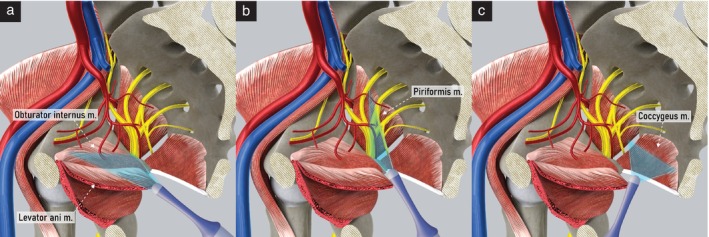
Ultrasound assessment of pelvic sidewall muscles. Schematic diagrams showing transvaginal approach to evaluate pelvic sidewall muscles: (a) obturator internus and levator ani muscles; (b) piriformis muscle; and (c) and coccygeus muscle. m., muscle.

1. The examination starts with visualization of the uterus in the midsagittal plane at the level of the internal cervical os, with the probe placed in the anterior fornix of the vagina.
2. The probe is rotated 90° and moved to the lateral vaginal fornix to visualize the uterosacral ligament, and then tilted laterally about 45° to follow the ligament laterally towards the pelvic sidewall.
3. With the probe directed dorsally, the obturator internus muscle appears just lateral to the uterosacral ligament, as a hypoechogenic band that covers most of the lateral wall of the lesser pelvis (Figure 3a).

#### Second step: identify the levator ani muscle

4 On the lateral side of the obturator internus muscle, a continuous hyperechogenic band is seen, corresponding to the body of the ischium. On the medial surface of the obturator internus muscle is the insertion of the levator ani muscle.
5 The levator ani (iliococcygeus) muscle is a triangular structure, whose lateral boundary is the tendinous arch of the obturator internus muscle. Medially, it converges towards the rectum. Its anterior border is the uterosacral ligament. Posteriorly, the coccygeus muscle and the anterior division of the internal iliac artery separate it from other anatomical structures (Figure 3a).

#### Third step: identify the piriformis muscle

6 After visualizing the obturator internus muscle, the probe is swept medially and dorsally to follow the obturator internus muscle and the hyperechogenic ischium until reaching the ischial spine, which represents its dorsal end. At that point, the structures crossing the greater sciatic foramen, including the piriformis muscle, can be visualized (Figure 3b).
7 Ventral to the piriformis muscle are the sacral plexus and branches of the anterior division of the internal iliac artery exiting the pelvis. Dorsally, the ala of the sacrum is seen as a hyperechogenic thin line bordering the medial part of the muscle.

#### Fourth step: identify the coccygeus muscle

8 The probe is returned to the anterior fornix, again visualizing the uterus in the midsagittal plane at the level of the inner cervical orifice.
9 The probe is moved behind the cervix at the posterior vaginal fornix. In the sagittal plane, the continuous hyperechogenic line of the sacrum and the coccyx can be visualized.
10 Tilting the probe slightly laterally, the parasagittal view is obtained, and the sacral foramina can be identified.
11 At the level of the fifth sacral vertebra, the probe is rotated 90° counterclockwise, and the fifth sacral vertebra and both coccygeus muscles appear in transverse section (Figure 3c).
12 Behind the coccygeus muscle, the sacrospinous ligament can be seen as a hyperechogenic thin line.

Examples of abnormal ultrasound findings related to the muscles of the pelvic sidewall are shown in Videoclip S6.

### Vessels

#### Anatomy

The common iliac artery arises from the abdominal aorta at the level of the fourth lumbar vertebra; it then courses downward and outward until the level of the sacroiliac joint, where it terminates by dividing into the external and internal iliac arteries. The external iliac artery runs along the superior border of the pelvic sidewall on the pelvic brim and becomes the femoral artery at the level of the inguinal ligament.

**Figure 4:**
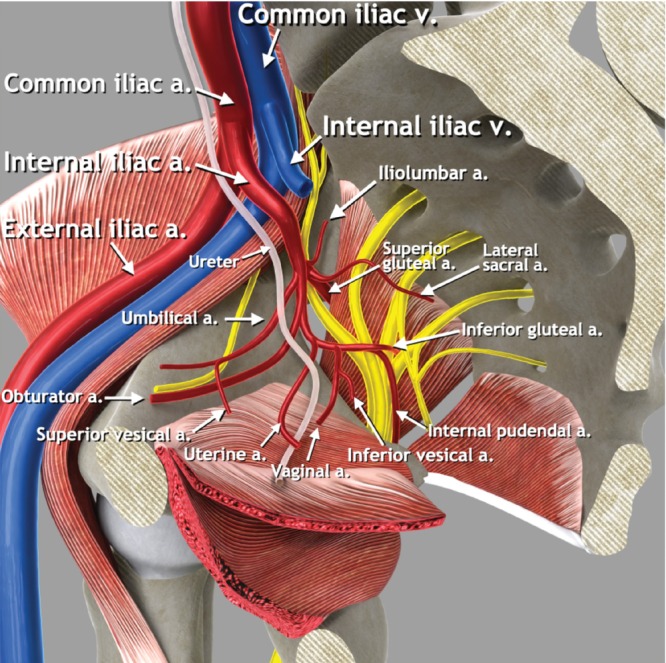
Pelvic sidewall vessels. Schematic diagram showing main pelvic sidewall vessels, with focus on anterior and posterior branches of the internal iliac artery. a., artery; v., vein.

**Figure 5:**
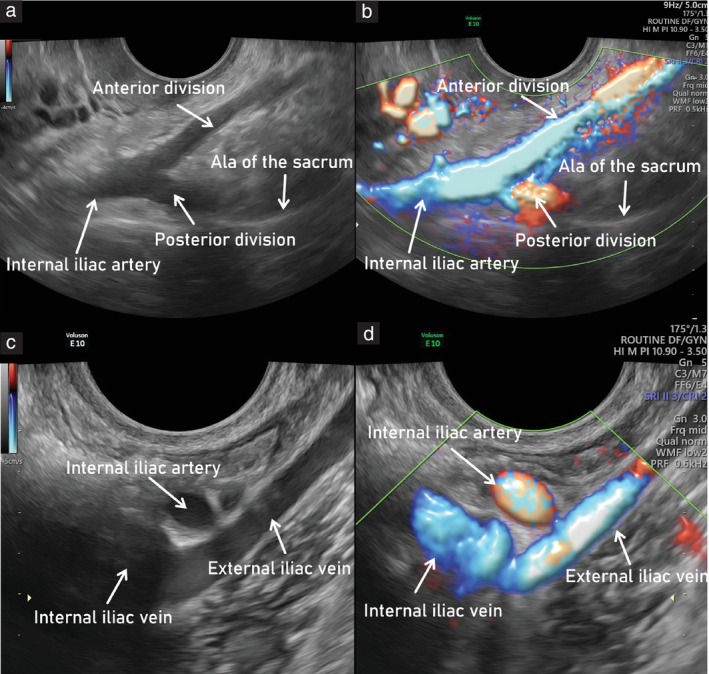
Main vascular bifurcations in the pelvis (left side). Ultrasound images showing: (a,b) branching of internal iliac artery into its anterior and posterior parts (forming a V shape) on grayscale (a) and power Doppler (b) assessment; and (c,d) interiliac bifurcation, showing external and internal iliac veins, on grayscale (c) and power Doppler (d) assessment. To obtain the entire course of the external iliac vessels, the probe should be rotated 90° from transverse (a,b) to longitudinal (c,d) plane.

The internal iliac artery courses caudally and dorsally to the upper margin of the greater sciatic foramen, where it divides into anterior and posterior branches (Figures 4 and 5). The anterior division gives rise to both visceral and parietal branches. The parietal branches are the inferior gluteal, internal pudendal and obturator arteries. The inferior gluteal artery usually passes the sacral plexus between the S2 and S3 roots and leaves the pelvis through the greater sciatic foramen together with the sciatic nerve, the internal pudendal vessels and the pudendal nerve, then travelling through the infrapiriform foramen inferior to the piriformis muscle (Figures 2 and 4). The internal pudendal artery curves around the ischial spine and sacrospinous ligament to enter the perineum through the lesser sciatic foramen. It passes through the ischioanal fossa in Alcock's canal, formed by a sheath of obturator fascia located on the medial wall of the obturator internus muscle, finally reaching the deep perineal pouch. The obturator artery runs anteroinferiorly over the pelvic wall, accompanied by the obturator vein and obturator nerve, being crossed by the ureter on the medial side and then entering the obturator foramen and obturator canal.

Visceral branches of the anterior division of the internal iliac artery include the uterine, vaginal, superior and inferior vesical arteries and middle rectal arteries, which arise consecutively and supply their respective organs, as suggested by their names. The most clinically relevant in the gynecological ultrasound setting is the uterine artery. This arises from the anterior division of the internal iliac artery and subsequently passes above the ureter, running between the sheaths of the broad ligament in the parauterine lymphovascular tissue (parametrium) and reaching the uterus approximately at the level of the internal cervical os, giving rise to the ascending and descending branches. The vaginal artery runs through the lateral parametrium, comprising the paracervix and paracolpium, and supplies the lower vagina, vestibular bulb and posteroinferior part of the bladder^7^, ^8^.

The posterior division of the internal iliac artery gives rise to the iliolumbar artery, the lateral sacral artery and the superior gluteal artery. The iliolumbar artery proceeds dorsally and superolaterally leaving the lesser pelvis as it ascends above the pelvic brim, before continuing its course in the greater pelvis. The lateral sacral artery proceeds towards the posterior pelvic wall and gives rise to the superior and inferior branches, which supply the sacral foramina. The superior gluteal artery runs between the lumbosacral trunk and the S1 nerve and leaves the pelvis superior to the piriformis muscle together with the superior gluteal nerve^9^. This is the only artery that leaves the pelvis through the suprapiriform foramen.

#### Ultrasound methodology (Videoclip S3)

#### First step: identify the uterine artery

1. The probe is placed in the anterior fornix of the vagina. From the midsagittal plane at the level of the internal cervical os, the probe is rotated 90° counterclockwise to obtain the transverse plane and to identify the uterine vessels, which appear as hypoechogenic bands.
2. Color Doppler or power Doppler can be used to differentiate blood vessels from the ureter on the lateral pelvic wall and to identify the branches of the internal iliac artery.

#### Second step: identify the branches of the anterior divisionof the internal iliac artery

3 Using color Doppler, the course of the uterine artery is followed laterally until the pelvic sidewall is reached. The uterine artery curves dorsally and cranially and meets with the obturator artery.
4 The obturator artery is seen running parallel to the obturator muscle, on the inner surface of the obturator internus muscle and the ischium.
5 Tilting the probe superiorly, the obturator vessels can be traced to the obturator canal. Near to the probe, the adductor longus muscle is visible in cross‐section.
6 Between the uterine and obturator arteries, in contact with the fundus of the urinary bladder, the superior vesical artery can be visualized.
7 Returning to the uterine artery and moving proximally along its curved course, its origin from the anterior division of the internal iliac artery is seen.
8 The branches of the anterior division of the internal iliac artery leaving the pelvis laterally through the infrapiriform foramen are the inferior gluteal and internal pudendal arteries. The inferior gluteal artery is thicker and runs between the nerves of the sacral plexus (S2–S3). The internal pudendal artery is directed towards the ischial spine and subsequently will enter Alcock's canal.

#### Third step: identify the branches of the posterior divisionof the internal iliac artery

9 Following its anterior division proximally along the anterior surface of the piriformis muscle, the branching of the internal iliac artery can be visualized (Figure 5).
10 At this level, two branches of the posterior division can be identified: the superior gluteal and lateral sacral arteries.
11 The superior gluteal artery runs along the anterior surface of the hyperechogenic ala of the sacrum and leaves the pelvis at the superior surface of the piriformis muscle, running towards the gluteus maximus muscle.
12 Medially, the lateral sacral artery runs under the sacroiliac joint on the surface of the ala of the sacrum and enters the anterior sacral foramen.
13 The lateral sacral artery can be followed back to its origin from the posterior branch of the internal iliac artery.

#### Fourth step: identify interiliac bifurcation and external iliac vessels

14 The anterior and posterior branches of the internal iliac artery are seen arising from the internal iliac artery bifurcation (forming a ‘V’ shape). The internal iliac artery is then followed up to the interiliac bifurcation with the external iliac artery, by moving the probe further, proximally and ventrally. The probe is still positioned in a transverse plane at this point in the examination (Figure 5).
15 In order to visualize the complete course of the external iliac artery up to the lacuna vasorum on both sides, it is then necessary to rotate the probe 90° towards the longitudinal plane.

Examples of abnormal ultrasound findings related to the vessels of the pelvic sidewall are shown in Videoclip S7.

### Lymph nodes

#### Anatomy

**Figure 6:**
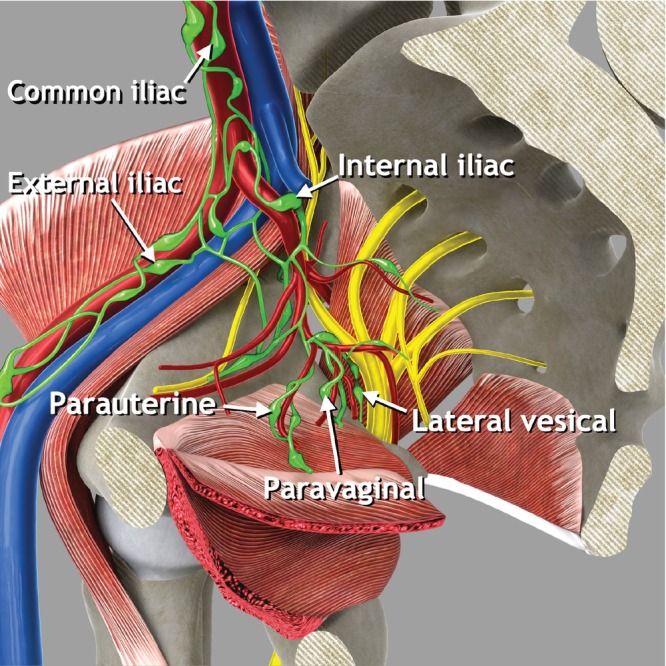
Pelvic parietal (iliac) and visceral lymph nodes. Schematic diagram showing pelvic parietal lymph nodes and some examples of pelvic visceral lymph nodes. Pelvic parietal (iliac) lymph nodes are divided into three groups: external iliac nodes (including obturator nodes), internal iliac nodes (including sacral nodes) and common iliac nodes. Some examples of visceral lymph nodes which can be assessed while evaluating the pelvic sidewall are shown: parauterine, paravaginal and lateral vesical lymph nodes.

The pelvic lymph nodes include parietal and visceral lymph nodes. The parietal (iliac) lymph nodes can be categorized into common iliac, external iliac and internal iliac (hypogastric) nodes, based on their position along the iliac arteries and veins^9^. In accordance with anatomical terminology, the obturator lymph nodes situated within the obturator fossa are considered as part of the external iliac lymph‐node group, whereas the sacral lymph nodes belong to the internal iliac lymph‐node group^10^, ^11^. The classification of pelvic visceral lymph nodes is based on their association with the pelvic organs (Figure 6). Lymph nodes adjacent to the bladder are referred to as lateral vesical, prevesical and postvesical lymph nodes. Those adjacent to the vagina, cervix and uterus are the paravaginal and the parauterine lymph nodes. The posterior pelvic compartment includes the pararectal lymph nodes, surrounding the rectum bilaterally, and the sigmoid visceral lymph nodes, located along the sigmoid colon. The visceral lymph nodes follow the course of the respective visceral vessels.

#### Ultrasound methodology (Videoclip S3)

1. Following the methodology described above to identify the vessels of the pelvic sidewall, the visceral and parietal (iliac) lymph nodes on the pelvic sidewall can also be identified.
2. Visceral lymph nodes, such as parauterine lymph nodes, drain the pelvic viscera and are located around visceral branches of the internal iliac artery (Figure 6).
3. Parietal lymph nodes are arranged around parietal branches of the internal iliac artery and external iliac vessels.
4. To visualize with high resolution both visceral and parietal lymph nodes of the pelvic sidewall, is important to combine the standard endocavitary approach (transvaginal or transrectal) with a transabdominal approach. Otherwise, there is a risk of overlooking infiltrated lymph nodes outside the field‐of‐view of the endocavitary probe, for example, those located ventrally to the external iliac vessels or close to the lacuna vasorum.
5. The Vulvar International Tumor Analysis (VITA) consensus recommended standard terminology to describe lymph nodes and for differentiation between benign and malignant nodes^12^.

Examples of abnormal ultrasound findings related to the lymph nodes of the pelvic sidewall are shown in Videoclip S8.

### Nerves

#### Anatomy

**Figure 7:**
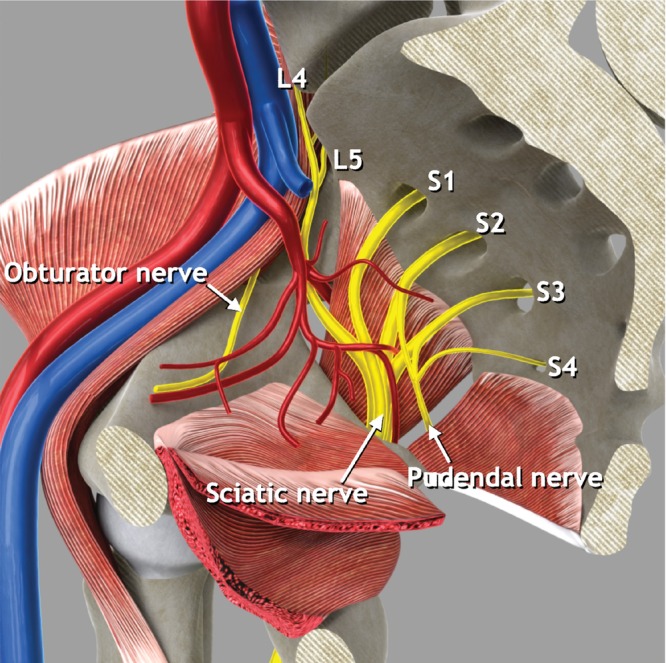
Pelvic sidewall nerves. Schematic diagram showing main pelvic sidewall nerves and nerve roots. L, lumbar nerve root; S, sacral nerve root.

The sacral plexus is composed of a network of fused nerve roots and is located on the anterior surface of the sacrum, on the posterior pelvic wall, ventral to the piriformis muscle and dorsal to the internal iliac vessels and ureter, running towards the greater sciatic foramen (Figure 7). This plexus is formed by the union of the anterior rami of the S1–S4 spinal nerves (sacral roots) and the descending lumbosacral trunk, which arises from the anterior rami of the L4 and L5 nerve roots^13^. The obturator nerve originates from three roots of the lumbar plexus (L2, L3 and L4). From the confluence of these three roots originates a single trunk that enters the pelvic cavity covered by the psoas major muscle. At the level of the pelvic inlet, it leaves the psoas and runs along the lateral wall of the cavity with the obturator vessels until it reaches the obturator canal. The pudendal nerve is formed by the union of three ventral rami (S2–S4) of the sacral plexus, converging adjacent to the pelvic sidewall. Along its course, the nerve is associated closely with the branches of the internal pudendal artery and vein. It exits the pelvis via the greater sciatic foramen, inferior to the piriformis muscle, and curves around the posterior aspect of the sacrospinous ligament. The sciatic nerve originates from the sacral plexus and is formed by fibers from all the nerves of the plexus (L4, L5, S1, S2 and S3). The nerve roots join to form a trunk adjacent to the sacrum. The sciatic nerve then exits the pelvis by passing behind the ischial bone, beneath the piriformis muscle, and laterally to the posterior cutaneous nerve of the thigh.

#### Ultrasound methodology (Videoclip S4)

Ultrasound assessment of the sacral plexus was described recently by Szabo *et al*.^14^, defining a new approach which enables rapid identification of this structure on transvaginal sonography.

#### First step: identify the sacral plexus

**Figure 8:**
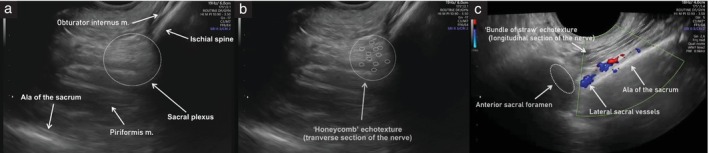
Ultrasound images of left sacral plexus. (a) Anatomic structures of the greater sciatic foramen with sacral plexus (dotted circle). (b) Transverse section of the nerves, exhibiting honeycomb‐like echotexture (dotted circles). (c) Longitudinal section of the nerves, exhibiting bundle‐of‐straw echotexture (dotted parallel lines); note, the sacral plexus can be traced up to the anterior sacral foramen (dotted oval) to identify the sacral nerve roots. m., muscle.

1. The probe is placed in the anterior vaginal fornix at the level of the internal cervical os, and rotated 90° until the transverse plane is obtained. The probe is then pointed towards the lateral vaginal fornix to identify the uterine vessels and the uterosacral ligament.
2. The uterosacral ligament is followed laterally up to the pelvic sidewall by tilting the probe laterally about 45°.
3. Maintaining this 45° tilt and following the uterosacral ligament dorsally, the obturator internus muscle appears just lateral to the uterosacral ligament, as a thin hypoechogenic band that covers most of the lateral wall of the lesser pelvis.
4. On the lateral side of the muscle, a hyperechogenic structure corresponding to the body of the ischium is seen.
5. The outer end of the probe is elevated about 45° and swept posteriorly, following the obturator internus and ischium to the ischial spine.
6. Transverse and oblique sections of the branches of the anterior division of the internal iliac vessels are seen.
7. Posterior to the vessels, hypoechogenic muscle fiber bundles with intervening echogenic perimysium, corresponding to the piriformis muscle, and a hyperechogenic line corresponding to the anterior surface of the sacrum, are visualized.
8. In longitudinal section, between the vessels and the piriformis muscle, the sacral roots of the sacral plexus can be visualized as hypoechogenic bands with echogenic septa (bundle‐of‐straw appearance). The transverse section of the nerves has a honeycomb‐like echotexture (Figure 8).

#### Second step: identify the sciatic and pudendal nerves

9 The sciatic nerve can be seen leaving the pelvis together with the inferior gluteal artery, inferior to the piriformis muscle.
10 The path of the pudendal nerve corresponds to the internal pudendal artery coursing around the ischial spine.

#### Third step: identify the sacral nerve roots

11 To visualize the sacral roots, the sacral plexus is traced medially, following the inferior gluteal artery back to the branching of the internal iliac artery. From there, the nerve is followed dorsally and medially until the sacral foramen is reached, and the sacral nerve roots and the lateral sacral artery can be identified.

#### Fourth step: identify the obturator nerve

12 The obturator nerve is seen as a thin hypoechogenic line with hyperechogenic epineurium following the course of the obturator artery, medial to the obturator internus muscle.

Examples of abnormal ultrasound findings related to the nerves of the pelvic sidewall are shown in Videoclip S9.

### Ureter

#### Anatomy

**Figure 9:**
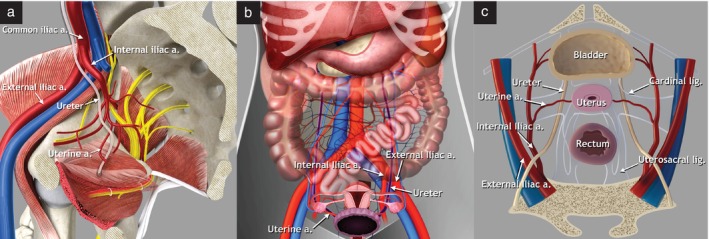
Abdominal and pelvic course of the ureter. Schematic diagrams demonstrating: (a) right hemipelvis, medial view, showing pelvic course of the ureter; (b) abdominal course of the ureter (purple), coronal view; and (c) pelvic course of the ureter, transverse view. a., artery, lig., ligament.

The ureter is a tubular structure that, based on its course, is divided into an abdominal part and a pelvic part (Figure 9). Originating from the kidney, it starts its path posterior to the renal artery and then follows along the anterior edge of the psoas major muscle. It then enters the pelvis at the pelvic brim where it crosses laterally to medially, anterior to the bifurcation of the common iliac artery and inferomedial to the ovarian vessels. It descends into the pelvis within a peritoneal sheath attached to the medial leaf of the uterine broad ligament and courses along the pelvic sidewall anterior and medial to the internal iliac artery. It runs along the lateral side of the uterosacral ligament. At the level of the internal cervical os, 1.5 to 2 cm lateral to the cervix, the ureter passes under the uterine artery, along the lateral side of the uterosacral ligament^15^. It continues its course through the lateral parametrium, which consists of the paracervix and paracolpium. The distal ureteric course divides the lateral parametrium into medial and lateral parts^7^. In its terminal part, the ureter passes within the ventral parametrium, dividing it into two portions. Medial to the ureter, the vesicouterine ligament can be identified cranially and the vesicovaginal ligament caudally. Lateral to the ureter, the lateral vesical ligament can be seen^2^, ^8^, ^16^. Finally, the ureter leaves the ventral parametrium, reaching the bladder at the posterolateral wall and entering near the trigone, where the ureters open into the bladder through the two ureteric orifices^17^.

#### Ultrasound methodology (Videoclip S5)

**Figure 10:**
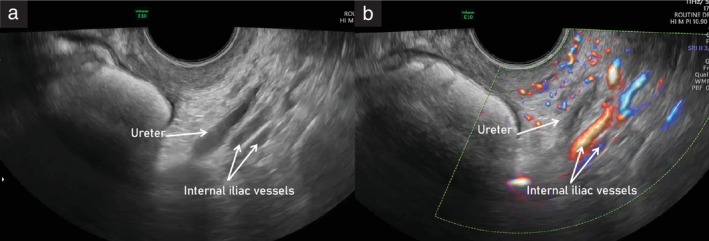
Ultrasound images showing course of the left ureter within the pelvic sidewall, running parallel to the internal iliac vessels, on grayscale (a) and power Doppler (b) imaging. The ureteric dilatation that can be observed in the grayscale image (a) is due to physiological peristalsis.

1. For a standardized assessment of the pelvic course of the ureters, it is usually easiest to start with visualization of the bladder trigone, with the bladder in longitudinal view. Then, withdrawing the probe slightly, while keeping it in the anterior fornix of the vagina, the midpoint of the trigone is visualized, where the urethra is seen exiting the bladder.
2. From the midsagittal plane, the probe is swept laterally on each side to identify the ureteric orifices (papillae). Then, by rotating and tilting the probe laterally, the intramural part of the ureter can be visualized as a tubular hypoechogenic structure entering the bladder trigone. The distal segment of the ureter will usually come into view once the probe is moved sufficiently towards the lateral pelvic wall^18^.
3. The distal part of the ureter passes through the anterior parametrium, surrounded by hyperechogenic connective tissue corresponding to the vesicovaginal ligament distally and the vesicouterine ligament proximally.
4. Using color Doppler, the uterine artery can be identified crossing the ureter in the lateral parametrium.
5. The probe is then rotated 90° to obtain the transverse plane and the ureter is followed along its course, parallel and ventral to the internal iliac vessels (Figure 10).
6. The ureter is then traced proximally, following its course under the peritoneum of the pelvic sidewall. The ureter can be distinguished from vascular structures by observing for peristalsis or using color Doppler.
7. In cases of parametrial infiltration (ventral/lateral/dorsal) or infiltration of the bladder trigone, ureteric obstruction may occur, and the resultant hydroureter and exact location of obstruction can be detected precisely using the high‐resolution endocavitary probe. However, if there is massive pelvic sidewall infiltration, the dilated proximal part of the ureter may be outside the field‐of‐view and may instead be better assessed via the transabdominal approach.
8. If there is suspicion of hydroureter, the ureteric diameter is measured in longitudinal section by placing the calipers on the outer borders of the ureter. To allow objective documentation, the distance between the site of obstruction and the vesicoureteral junction should also be measured.

Examples of abnormal ultrasound findings related to the ureter are shown in Videoclip S10.

## CLINICAL RELEVANCE OF THE PELVIC SIDEWALL

Accurate identification of the muscles, vessels, nerves, ureters and other structures of the pelvic sidewall enables assessment of their involvement in malignant and benign gynecological pathology, for example, in the case of advanced gynecological cancer, deep endometriosis or rarer pathologies such as intravascular leiomyomatosis.

In the field of gynecological oncology, sonographic assessment of the pelvic sidewall plays a key role in the locoregional staging of cancer, detection of recurrence, evaluation of resectability and preoperative multidisciplinary team and surgical resource planning in this anatomical region. For example, in cervical cancer, tumor involvement of the pelvic sidewall is considered present when the tumor extends to within 3 mm of the obturator internus or piriformis muscle, with simultaneous loss of fat planes, or when there is resultant hydronephrosis or a non‐functioning kidney^19^. This upstages the disease from FIGO (International Federation of Gynecology and Obstetrics) Stage IIB to Stage IIIB^20^. In recurrent disease, the involvement of the pelvic sidewall confers a poorer prognosis compared with central pelvic recurrence and may necessitate a laterally extended endopelvic resection in a previously irradiated pelvis.

**Figure 11:**
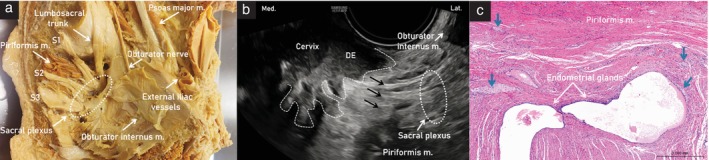
(a) Cadaveric specimen of left hemipelvis, showing sacral roots (S) and sacral plexus (dotted oval), leaving the pelvis through the greater sciatic foramen. (b) Ultrasound image showing left sacral plexus (dotted oval) infiltrated by a nodule of deep endometriosis (DE) with spiculated margins (dashed line) developing laterally from the cervix. Fibers of the sacral nerves are indicated (black arrows). (c) Corresponding histological image following laparoscopic excision of the lesion, showing perineural endometriosis: the endometrial glands are surrounded by connective tissue and stroma, nerve fibers are indicated (blue arrows) and striated muscle fibers (piriformis muscle) are visible in upper part of the specimen. Lat., lateral; m., muscle; Med., medial.

Transvaginal ultrasound is also considered a first‐line imaging technique in benign conditions such as endometriosis, which affects approximately 5–10% of women worldwide^21^. Deep infiltrating endometriosis in the pelvic sidewall along the course of the ureter is rare, but, if unrecognized, may lead to silent loss of kidney function on the affected side^22^. Occasionally, endometriosis may also involve the sacral plexus, which is the most common neurological manifestation of deep infiltrating endometriosis, followed by infiltration of the sciatic nerve (Figure 11)^23^. Diagnosis of endometriosis causing neurological sequelae is challenging and requires a comprehensive methodological approach to assessment of the pelvic sidewall. Without such an approach, there may be delays that prevent the prompt direction of patients to the most appropriate treatment^1^.

**Figure 12:**
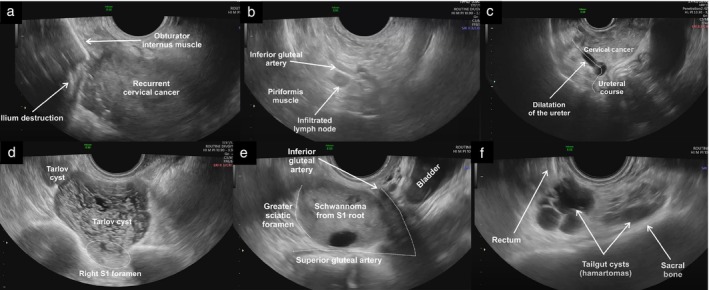
Ultrasound images illustrating various pathologies involving the pelvic sidewall. (a) Recurrent cervical cancer extending laterally on the right side, disrupting part of the obturator internus muscle and the iliac bone; (b) infiltrated pelvic parietal lymph node, which appears rounded with loss of normal sonographic architecture due to infiltration by cervical cancer, located along the course of the internal iliac branches; (c) right hydroureter due to locally advanced cervical cancer infiltrating the proximal third of the ureteric course; (d) Tarlov (perineural) cysts protruding from the right anterior sacral foramen of the first sacral vertebra (S1), presenting as multilocular cystic masses with thin septations; (e) pelvic‐nerve‐sheath tumor (Schwannoma) arising from the S1 sacral root and protruding into the greater sciatic foramen, presenting as a solid lesion with cystic areas; and (f) tailgut cysts (cystic hamartomas) located along the anterior surface of the sacral bone, presenting as multiloculated cystic masses with low‐level intracystic fluid (mucoid content).

Finally, the capacity to assess accurately this anatomical region could also lead to improved detection and diagnosis of non‐gynecological pathologies that may be confused with gynecological tumors, such as Tarlov cysts and peripheral‐nerve‐sheath tumors, reducing the risk of unnecessary or even harmful surgical procedures or interventions (Videoclips [Link], [Link], [Link], [Link], [Link], [Link], Figure 12)^4^.

## FUTURE DIRECTION (TRAINING)

The previous standard tool for the preoperative evaluation of lesions affecting the pelvic sidewall was pelvic magnetic resonance imaging (MRI). However, recent research has identified ultrasound as a viable alternative to MRI in the assessment of the pelvic sidewall. A new field of transvaginal sonography, termed pelveoneurosonography, has highlighted the possibility of visualizing the pelvic nerves on ultrasound^14^. For individualized treatment of both benign and malignant gynecological disease, the combination of both surgical and sonographic skills in one person (surgeon‐sonologist) with advanced knowledge in both fields is fundamental. This applies both to gynecological oncologists dealing with laterally extended pelvic tumors and gynecologists from referral centers managing deeply infiltrating endometriosis. Therefore, we strongly believe that education and training in ultrasound assessment of the pelvic sidewall should be embedded within the surgical curriculum and that this will improve patient care.

## CONCLUSION

In this methodological consensus opinion, we present a step‐by‐step standardized approach for transvaginal or transrectal ultrasonographic evaluation of anatomical structures within the pelvic sidewall, and define useful sonographic landmarks. This multicenter collaboration of expert gynecologists and gynecological oncologists, supported by online videoclips obtained during specialized gynecological ultrasound examinations at a tertiary‐level center, demonstrates that assessment of the pelvic sidewall is achievable with this approach.

## Supporting information


**Videoclip S1** Dynamic tests of mobility.

**Videoclip S2** Muscles of the pelvic sidewall (demonstration of normal anatomy).

**Videoclip S3** Vessels and lymph nodes of the pelvic sidewall (demonstration of normal anatomy).

**Videoclip S4** Nerves of the pelvic sidewall (demonstration of normal anatomy).

**Videoclip S5** Course of the ureter (demonstration of normal anatomy).

**Videoclip S6** Muscles of the pelvic sidewall (demonstration of clinical cases).

**Videoclip S7** Vessels of the pelvic sidewall (demonstration of clinical cases).

**Videoclip S8** Lymph nodes of the pelvic sidewall (demonstration of clinical cases).

**Videoclip S9** Nerves of the pelvic sidewall (demonstration of clinical cases).

**Videoclip S10** Ureter (demonstration of clinical cases).

**Videoclip S11** Demonstration of other clinical cases.

## Data Availability

Data sharing not applicable to this article as no datasets were generated or analysed during the current study.
